# Supplementary material for: Operon mRNAs are organized into ORF-centric structures that predict translation efficiency
Source: eLife. 2017 Jan 31;6:e22037. doi: 10.7554/eLife.22037 (PMC5318159; doi:10.7554/eLife.22037)
Supplement: Figure 4—source data 1. — A multiple linear regression model is applied to predict TE based on the following features: mRNA structure level of various portions of the ORF, codon usage predicted by tAI (dos Reis et al., 2004; Tuller et al., 2010), codon influence metric (Boël et al., 2016), and the strength of Shine-Dalgarno sequence (using the RBS Calculator established by Salis et al from https://github.com/hsalis/Ribosome-Binding-Site-Calculator-v1.0). (A) Comparison between the experimentally measured TE and the model-predicted TE. The red dashed line indicates the y = x diagonal line. (B) Relative contribution of the factors in predicting TE, calculated from stepwise regression. Y-axis: R2 of different models with stepwise addition of individual factors. Asterisks indicate significant improvement of model (based on ANOVA, with significance codes: 0 ‘***’ 0.001 ‘**’ 0.01 ‘*’ 0.05). DOI: http://dx.doi.org/10.7554/eLife.22037.009 [file elife-22037-fig4-data1.docx]

**Figure 4 - figure supplement 3**

**Linear Regression Model**

To build a comprehensive model for TE prediction, we chose ORFs passing the following criteria: (1) ORFs are more than 200nt long to better separate the head and the body portions of ORF; (2) There is ≥ 20 nucleotides separating from the upstream ORF (3) ORFs have ≥ 15 reads/nt of WT *in vivo* DMS-seq signal. The total number of selected ORFs is 822.

We applied a multiple linear regression model to predict TE that is based on the following features: mRNA structure level (Gini index) of various portions of the ORF, codon usage predicted by tAI (dos Reis et al., 2004; Tuller et al., 2010), codon influence metric (Boel et al., 2016), and the strength of Shine-Dalgarno sequence (using the RBS Calculator established by Salis et al from https://github.com/hsalis/Ribosome-Binding-Site-Calculator-v1.0). The coefficients of factors are shown in the model equations, with significance indicated. To avoid overfitting, 5-fold cross validation (CV) was applied to models, and the R^2^ values after CV were compared to the adjusted R^2^ values calculated from the model using R package “caret” (http://topepo.github.io/caret/index.html). Analyses of the model are shown in two figures: (A) Comparison between experimentally measured TE to model-predicted TE; (B) Relative contribution of the factors tested in the model in predicting TE, calculated using stepwise regression.

For stepwise regression, we used forward selection to compare the change of R^2^ value after adding each variable sequentially to the model and for each step chose the factor that improves the model the most. The results of stepwise regression for the model are shown below. Consistent with results from pairwise correlations, mRNA structure level plays a much more important role than the codon usage predicted by tAI or codon influence. Inclusion of the latter has marginal improvement in the predictive power. The importance of mRNA structure level depends on the region included in the calculation: we find increasing effect as larger regions of the ORF are considered in the structural analysis, and the ORF-wide mRNA structure is predominant among the factors tested.

$${log}_{2}TE=5.93 -1.66\times Gini(head)-15.6\times Gini(body)+2.73\times tAI+0.23\times CI+0.03\times aSD$$

(1) Gini(head) ***: Gini index of ORF head (from 20nt upstream to 60nt downstream of start codon) calculated from *in vivo* WT DMS-seq data

(2) Gini(body) ***: Gini index of ORF body (from 60nt downstream of start codon to stop codon) calculated from *in vivo* WT DMS-seq data

(3) tAI **: tRNA adaptation index

(4) CI ***: codon influence metric

(5) aSD **: strength of Shine-Dalgarno sequence

(Significance codes: 0 ‘***’ 0.001 ‘**’ 0.01 ‘*’ 0.05)

Results:

Adjusted R^2^ calculated from model: 0.611

5-fold Cross Validation R^2^ (seed = 100): 0.609 ± 0.050


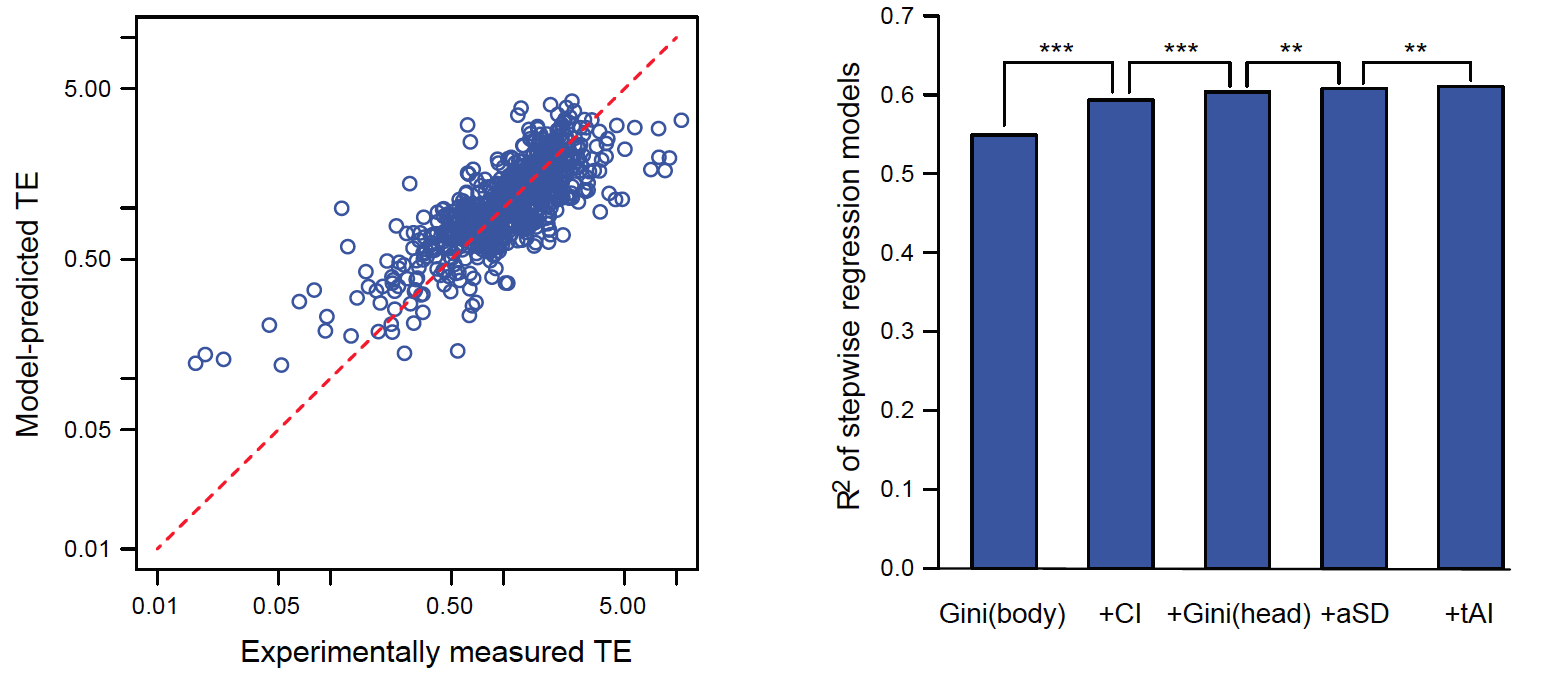


Figure B. Relative contribution of the factors in predicting TE, calculated from stepwise regression. Y-axis: R^2^ of different models with stepwise addition of individual factors. Asterisks indicate significant improvement of model (based on ANOVA). Significance codes: 0 ‘***’ 0.001 ‘**’ 0.01 ‘*’ 0.05

Figure A. Comparison between the experimentally measured TE and the model-predicted TE. The red dashed line indicates the y = x diagonal line.
